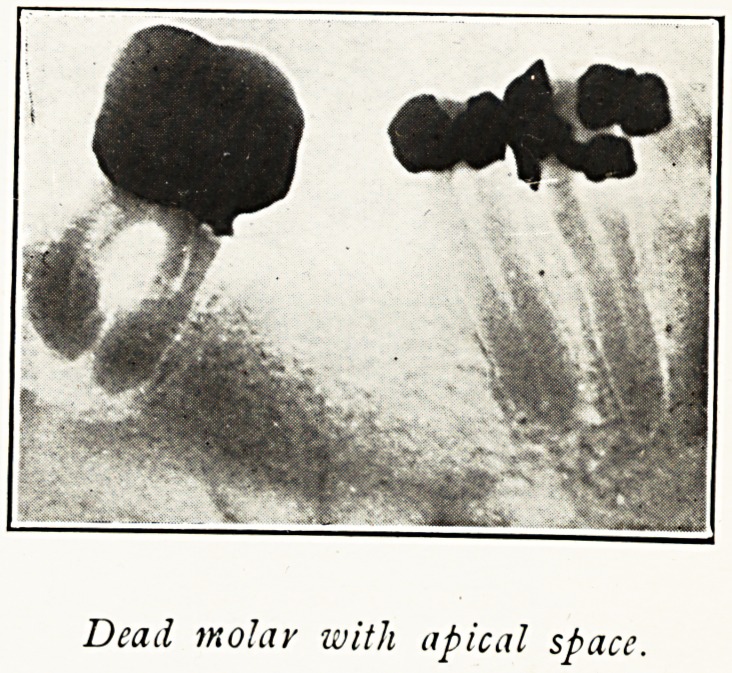# Oral Sepsis as a Source of Systemic Infections
1Introduction to a discussion at the meeting of the Bristol Medico-Chirurgical Society held on 8th March, 1922.


**Published:** 1922-09

**Authors:** W. R. Ackland

**Affiliations:** Dental Surgeon, Bristol Royal Infirmary; Lecturer and Examiner in Dental Surgery, University of Bristol; President of the Odontological Section of the Royal Society of Medicine


### ORAL SEPSIS AS A SOURCE OF SYSTEMIC INFECTIONS.[1]

BY

W. R. Ackland, M.R.C.S., L.D.S., M.D.S.,

*Dental Surgeon, Bristol Royal Infirmary; Lecturer and Examiner in Dental Surgery, University of Bristol; President of the Odontological Section of the Royal Society of Medicine.*

---

In introducing this discussion my object is to get from my colleagues in the various departments their opinions and experiences of the part oral sepsis may play in producing the special ailments which interest them.

We want information from the ophthalmologist, the ear, nose and throat surgeon, the physician, the skin and mental specialists, and the general practitioner, and I may get a rebuke from the gynæcologist! I should like, in fact, to pool all our knowledge and try and strike a sane average of the extreme and divergent views which are held on this subject.

On the one hand, we have men who attribute every mortal ailment to oral sepsis, even sterility in females! On the other, those who, having met many patients with septic mouths and apparently none the worse, refuse to take it into account at all.

*Oral Sepsis* appears to be the product of any or all of several conditions: 1, Neglect of the teeth, whether natural or artificial—in short, want of cleanliness; 2, Caries;

[1] Introduction to a discussion at the meeting of the Bristol Medico-Chirurgical Society held on 8th March, 1922.

*Normal gum, showing "high tide" of bone.*

*Pyorrhœa, showing "low tide" of bone.*

*Dead teeth with apical spaces.*

*Dead molar with apical space.*

3, Dead teeth ; 4, Pyorrhœa ; 5, Pathological conditions of the mucous membrane generally. It is obvious that the first may lead to all the others.

*How do we account for the divergence of opinion as to its effects?*

The answer is, that the effects vary out of all apparent proportion to the cause, so I will put the question in another way.

*Why does a small amount of Oral Sepsis occasionally cause such ill-effects in some people, while others with badly septic mouths escape?*

Here we open up the subject of vital resistance, and I shall hope to hear something about this in our discussion. I recognise in some of these cases the sort of immunity foreigners enjoy when the drainage is either bad or absent. You or I go there and we get typhoid at once! Horder says the ill-effects depend upon the net amount of toxin absorbed by the circulation, and not on the gross amount of toxin formed at the seat of infection.

The measure of resistance to or immunity from the ill-effects of oral sepsis is apparently directly proportionate to the preponderance of the forces of defence over those of attack, and this varies with health, and is diminished with advancing years. For instance, we constantly see patients with neglected mouths who keep perfectly well till strain or illness affects them. We see others whose neglect is not punished till middle age comes on.

On the other hand, it may be argued that probably the mouth condition was a predisposing cause of illness or breakdown, and that middle age with its diminishing forces is hastened by oral sepsis.

*The method of infection* is by either or both of two channels: 1, the intestinal canal; 2, the blood stream.

In a healthy stomach the gastric juice kills the germs, but in cases where the gastric juice is diminished in quantity or in acidity the germs pass on and find a favourable medium in the small intestine.

The blood stream is affected either directly or through the lymphatics.

*Diseases arising from Oral Sepsis* :—

1. Carcinoma of tongue, palate or cheek.

2. Gastro-intestinal troubles : (*a*) Gastritis ; (*b*) Duodenitis ; (*c*) Catarrh of bile duct ; (*d*) Appendicitis ; (*e*) Colitis.

3. Ear, nose and throat cases : (*a*) Suppuration of antrum ; (*b*) Tonsillitis ; (*c*) Pharyngitis, Eustachian and middle ear trouble, tinnitus ; (*d*) Laryngitis.

4. Eye cases : (*a*) Iritis ; (*b*) Choroiditis ; (*c*) Neuro-retinitis, etc.

5. Skin troubles : (*a*) Pemphigus ; (*b*) Alopecia ; (*c*) Rosacea ; (*d*) Acne ; (*e*) Erythemata ; (*f*) Urticaria ; (*g*) Eczema.

6. Lymphatic affections : (*a*) Acute and chronic lymphadenitis ; (*b*) Angina Ludovici.

7. General conditions or diathesis due to bacterial intoxication : (*a*) Chronic toxæmia ; (*b*) Chronic rheumatism, synovitis, arthritis, lumbago, myalgia, fibrositis ; (*c*) Anæmia ; (*d*) Arterio-sclerosis.

8. Conditions due to absorption of infection in the mouth : (*a*) Malignant endocarditis ; (*b*) Septicæmia ; (*c*) Neuritis ; (*d*) Nephritis.

*Diseases influenced by Oral Sepsis* :—

There is no doubt that all diseases are profoundly modified by oral sepsis. Various eye troubles especially, but also the exanthematous fevers, pneumonia, typhoid. I expect, too, to hear from the mental specialists present that they recognise it as a factor in many of their patients.

The foregoing list, which I have culled from various sources apart from my own cases, represents groups of marked conditions. But there are various minor conditions or symptoms scarcely belonging to any of these groups which it is most important to recognise.

For instance, the earliest symptoms of systematic disturbance in a recent case of pyorrhœa were : 1, Lassitude, a sense of unfitness ; 2, Headache on rising in the morning, which disappeared as the day went on ; 3, Bad taste in the mouth ; 4, Follicular ulcers in mouth ; 5, Loss of appetite ; 6, Loss of weight ; 7, Evening rise of temperature ; 8, Cardiac irregularity ; 9, Shortness of breath ; 10, Mental confusion and indecision ; 11, Insomnia.

Every symptom disappeared within a month after complete extraction.

We say there is no real cure for pyorrhœa, and when general symptoms supervene extraction is the only remedy. Yet I have several cases under my care where no such symptoms have occurred.

Perhaps the most valuable treatment is a course of violet rays, together with a neo-chlorine wash, which the patient uses after meals. Should any tooth get annoyingly loose or tender I extract it at once, and if general symptoms occur I take a skiagram of all the teeth and remove every suspicious one.

*Artificial dentures causing Oral Sepsis :—*

A very interesting case of loss of health due to ulceration caused by an ill-fitting plate was sent me by Dr. Frank Crossman. I have seen several of these cases, and the practitioner should be on the look-out for them.

And while I am referring to dentures let me put the following on record. I made a suction upper denture for a patient. I cannot tell you whether I warned him to take it out daily and clean it—you would not think such a warning necessary ; but he was seriously ill in a year or two, showing all the symptoms of severe infection, without any apparent cause. His doctors could make nothing of it till they discovered the plate. He had never taken it out. There was a perforating ulcer of the palate under it, and it cost him his life. Oral sepsis with a vengeance !

But my most interesting cases have been due not to pyorrhœa or superficial sepsis, but to deep sepsis at the apex of the roots of dead teeth. Suppuration without a chance of drainage is " bottled up," so to speak, in the depths of the bone, often without pain or local symptoms. The infection in these cases goes into the circulation direct. Horder points out the special streptococcus (*s. salivarius*) found on these roots has been isolated by him from the blood in certain cases of endocarditis. He further says that he associates with dead teeth most of those cases of chronic inflammation of fibrous tissues and serous membranes, rheumatism in its various forms, myalgia, fibrositis, and neuritis.

The question of the advisability of keeping dead teeth at all is being actively discussed. Even the American dentists are " shying off " crowns and bridges, which depend so largely for their support on teeth whose nerves have been destroyed. For my own part I keep an open mind, and am not in too great a hurry to condemn such teeth without the positive evidence of a skiagram.

*Eye cases.*—I have had some most interesting eye cases due to dead teeth :—

1. Central colour scotoma, due to dead canine, cured by extraction.

2. Difference in colour of eyes, of fourteen or fifteen years' standing. Discoloration of left eye, due to dead lateral incisor. Colour regained after extraction.

3. Commencing neuro-retinitis cured by extraction of dead lateral incisor.

4. Various cases of impairment of vision, caused by dead roots, removal of which was followed by immediate improvement.

*Mental cases.*—The dental treatment of these cases is naturally full of difficulties. Toothache calls for immediate treatment, and the patient may submit with some degree of reasonableness. But the systematic examination and filling of teeth must be almost impossible with some of them. I imagine, too, the tooth brush is not as properly used as it ought to be, with the result that oral sepsis is more often present in the insane than in ordinary patients. The chronic toxæmia accentuates the mental depression, and on this account alienists lay great stress on the importance of dental treatment.

*Rheumatism.*—I have met with several disappointments in treating cases of rheumatism associated with pyorrhœa by wholesale extraction. Indeed, I have been led to think that the pyorrhœa was the result of rheumatism, and not the cause. In my experience it is more often associated with dead teeth and stumps.

*Neuritis* of dental origin is practically always connected with apical mischief in dead teeth.

*Arthritis* has in my experience been mostly due to dead teeth.

### CONCLUSIONS.

My early cases occurred before the dangers of oral sepsis were known, and I am bound to say some of my best results were obtained by good luck, and not as a result of diagnosis.

As I look back I remember finding myself frequently disappointed in the result of cases which appeared to be similar in origin.

I remember many cases of pyorrhœa with general symptoms in which extraction did not clear up the trouble, and so I began to realise that the mouth was only one of many areas which may be the source of infection.

I have made up my mind that pyorrhœa is sometimes an effect of a general condition, and not the cause of it.

It is a frequent sequel of malaria and mercurial salivation, and a concomitant of chronic nephritis and rheumatism. Possibly a " vicious circle " is created in some of these cases, and cause and effect are mixed.

Dead teeth and stumps are more harmful in my experience than pyorrhœa.

Pyorrhœa may cause general systemic conditions—malaise, fever, loss of weight, insomnia.

Dead teeth cause unexpected effects in odd corners of the anatomy—synovitis of an ankle joint or knee, neuritis of one arm, iritis, ulcerative endocarditis, tinnitus, deafness, varicose veins, and apparently in one patient—sterility !

## Figures and Tables

**Figure f1:**
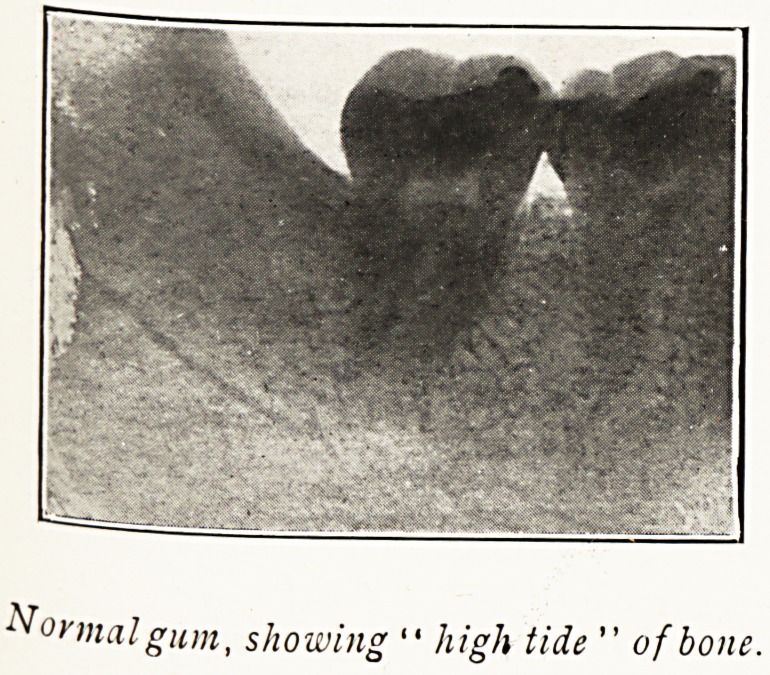


**Figure f2:**
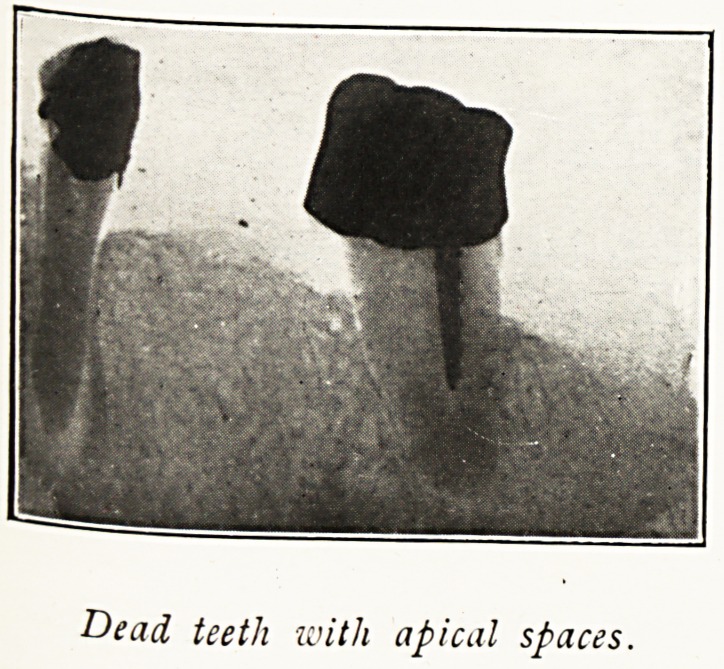


**Figure f3:**
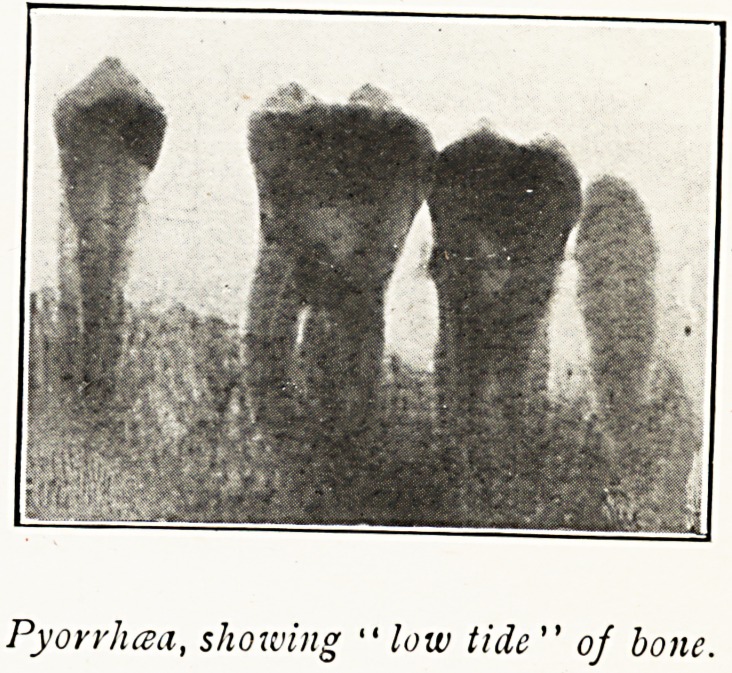


**Figure f4:**